# Addressing the Social Determinants of Health in South Korea: Moderating Role of mHealth Technologies

**DOI:** 10.3390/ijerph19031871

**Published:** 2022-02-07

**Authors:** Woohyun Yoo

**Affiliations:** Department of Mass Communication & Institute of Social Sciences, Incheon National University, 119 Academy-ro, Yeonsu-gu, Incheon 22012, Korea; wyoo@inu.ac.kr

**Keywords:** mHealth, mobile health technology, social determinants of health, health inequality, health self-efficacy, health status

## Abstract

Mobile health (mHealth) technologies may reduce or widen health inequalities. Despite the extensive literature in support of both of these contrasting views, little attention has been paid to the role of mHealth technologies with regard to social strata and health in the context of South Korea, a country with one of the highest usages of smartphones worldwide. This study examined the effects of social determinants on health self-efficacy and health status and explored how mHealth technologies moderate the impacts of social determinants on health outcomes. Data were collected via online surveys from 29 July to 3 August 2021. Survey data from 1187 Korean adults showed that men had higher levels of health self-efficacy than women. The higher an individual’s education level, the greater their subjective health status. Individuals with higher levels of monthly household income, social capital, and healthcare quality reported higher levels of health self-efficacy and superior health status. The use of mHealth technologies moderated the associations between social determinants and health outcomes. Specifically, monthly household income and social capital had smaller effects on health self-efficacy and health status among those who used higher levels of mHealth technologies. Among higher users of mHealth technologies, females reported better health status than males, while men showed better health status than women in the low-user group. These findings highlight the effectiveness of mHealth technologies in addressing health disparities.

## 1. Introduction

Health inequalities are recognized as a major public health issue through the world. The World Health Organization (WHO) Commission on Social Determinants of Health (CSDH) calls on governments to pay closer attention to public health policy, practices, and research regarding the social determinants of health (SDH) to reduce health inequalities and improve the health of the most vulnerable and disadvantaged groups [1]. SDH describes the conditions in which people are born, grow, live, work, and age, which are shaped by the distribution of money, power, and resources [1]. Specifically, they include the factors of socioeconomic status, education, neighbourhood and physical environment, employment, social support networks, and quality of healthcare [2].

SDH have significant effects on health. For example, outdoor air pollution exacerbates asthma symptoms [3] and depression [4]. Levels of income and education also determine a variety of health outcomes, such as chronic diseases, psychological well-being, and life expectancy, across the lifespan [5,6,7,8]. Healthcare systems, which dictate access to healthcare and quality of care, cause differences in life expectancy and infant mortality [9,10]. Social capital is an important determinant of population health. Although it is a widely used and multifaceted concept applied in a range of fields, social capital generally means the process through which social support is exchanged in the domain of public health [11]. Social support is defined as the networks of family, friends, neighbours, and community members that are available in times of need to provide psychological, physical, and financial support [12]. The literature indicates that social support is an important cause of various health outcomes [13,14,15,16]. Regarding social, environmental, and economic factors, gender is also a powerful determinant of health. Gender is the term for the set of socially constructed roles, norms, behaviours, activities, and attributes that require men and women to take on different positions in various social contexts [17]. In many societies, these different social constructions privilege men over women, producing gender inequalities that disproportionately influence women’s health. For this reason, women experience higher levels of psychiatric disorders and chronic illness and more functional limitations over their course of life than men [18,19,20].

Addressing the SDH is critical for reducing health disparities and achieving health equity. A growing number of researchers, scientists, and policy makers have focused on how to address the SDH to achieve better population health and reduce inequality. Mobile health (mHealth) technologies have emerged as a promising solution that can reduce the health inequality caused by the SDH. mHealth refers to mobile and wireless applications, including text messaging, apps, wearable devices, and remote sensing in the delivery of health-related services [21]. mHealth services enable people to take care of their own health or treat a condition using their own smartphones [22]. As ownership of smartphones, which allows people to access to mHealth services through apps, continues to grow, mHealth technologies are becoming more and more accessible. Accordingly, mHealth technologies can help reduce health disparities by disproportionately improving the health and well-being of vulnerable populations. mHealth technologies show promise in engaging patients in self-management, health-promotion behaviours, and chronic disease management [23,24]. Given their high use across socioeconomic groups, including low-income, low-literacy groups, and racial/ethnic minorities, mobile technologies represent a natural bridge across the digital divide to serve typically difficult-to-reach populations. In support of this suggestion, there is growing evidence that the digital divide continues to narrow [25].

mHealth programs can also meet the needs of vulnerable populations in terms of cost and personnel. For example, interventions utilizing Short Message Service (SMS), or text messages, allow healthcare providers and patients to communicate outside of regular office visits. This may be particularly attractive for socially complex or vulnerable patients in under-resourced communities. A systematic review of studies on mHealth tools for vulnerable populations found that mHealth interventions improve health outcomes, including supporting weight loss and increasing self-efficacy for health knowledge [26]. In addition, mHealth applications and text-messaging programs can provide a sense of social support among vulnerable populations suffering from chronic diseases. Social support enables health-promoting behaviours and is conducive to improved health outcomes among diverse vulnerable populations [27]. Mobile social support interventions reduce the need for physical presence and make social support interventions more accessible to vulnerable populations in need. Previous studies have found that mHealth-based social support interventions are practicable, acceptable to medically underserved patients with diabetes, and result in positive outcomes for diabetes-specific outcomes [28,29].

However, mHealth technologies also have the potential to widen health disparities. Although the use of mobile phones and smartphones is widespread, people with limited technological skills may be reluctant to use technology to obtain health information on their phones. Namely, there exists a second-level digital divide indicating differences between groups of people in terms of the skills needed to effectively utilize mobile technologies. Previous studies have shown that sociodemographic and socioeconomic characteristics are significant determinants of digital health-related behaviours [30,31]. In addition to skills and use of digital technologies (second-level divide), research on the digital divide has begun to focus on who benefits most from technology use (third-level divide). The third-level digital divide concerns disparities in the benefits that an individual obtains from different access (first-level) and different use (second-level) of digital technologies [32]. The third digital divide occurs in the health domain as well. For example, Neter et al. [33] found that individuals engaged in more health-related Web 1.0 activities used more healthcare services and reported greater perceived outcome of health-related internet use. Chiu and Li [34] showed that the association between socioeconomic status and digital skills results in disparities in food risk prevention. Thus, this digital divide plays a critical role in exacerbating existing health inequalities.

The requirement to use unfamiliar features of mobile phones can also exclude populations from the benefits of mHealth technologies, thereby worsening the digital divide [35]. This makes it challenging for many people to maintain a continuous and reliable wireless connection to the internet, which would severely limit their ability to benefit from mHealth applications [36]. The existence of a digital divide is sufficiently well-attested and reduces opportunities for disadvantaged individuals. A survey conducted in the United States indicated that cost was the greatest barrier to owning a mobile device among a predominantly African American sample of low-income individuals with serious mental health problems [37]. On the other hand, access does not guarantee benefit from mHealth technologies. Difficult or unfamiliar user interfaces may deter people with a lower socioeconomic status from making effective use of mobile technologies for their health. Significant gaps in the trust of health information from internet sources has been observed across low-income and ethnic groups [38]. It is likely that these digital inequalities and lack of trust in online health information will significantly limit the effectiveness of mHealth technologies for enabling minority and low-income individuals to benefit by self-diagnosing acute symptoms or tracking and managing chronic health conditions. Thus, significant advances in mHealthcare systems spread slowly and acquire lower rates of utilization within vulnerable populations, thus further exacerbating disparities in healthcare and health outcomes among such groups.

Although a large body of literature exists to support these two contrasting perspectives on mHealth’s effects on health inequalities, insufficient attention has been paid to the role of mHealth technologies between social strata and health in the context of South Korea, which features among the highest smartphone use rates in the world. According to a Pew Research Center survey of mobile technology adoption around the globe [39], nine out of ten South Koreans owned a smartphone, marking the highest level of smartphone ownership among 27 countries surveyed. Smartphone users install more apps than users of other mobile devices [40]. In accordance with the high rates of smartphone and app usage, mHealth is rapidly gaining attention in the field of health promotion, prevention, healthcare, and social support in South Korea.

**Figure 1:**
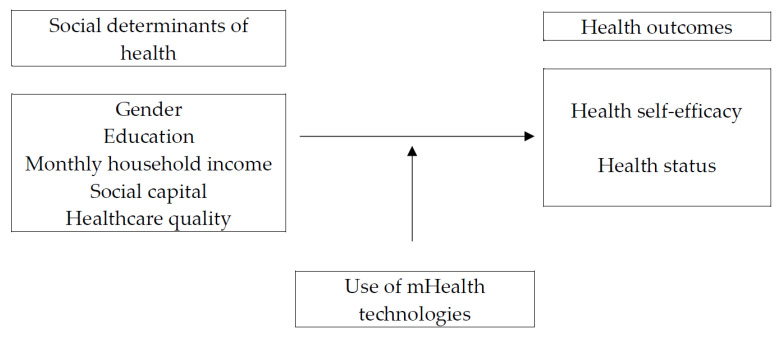
Research framework.

To address this gap in the literature, the current study explores the potential of mHealth technologies to address the SDH in the context of South Korea. Specifically, this research pursues two objectives. The first is to investigate whether the SDH have an influence on health. The second is to examine whether mHealth technologies moderate the effects of the SDH on health. In brief, the following hypotheses and research questions are proposed (see Figure 1). (H1) Males will have (a) higher health self-efficacy and (b) better health status than females. (H2) Higher education, (H3) higher monthly household income, (H4) higher social capital, and (H5) higher healthcare quality will be related to (a) higher health self-efficacy and (b) better health status. (RQ1) How does the use of mHealth technologies moderate the association between the SDH and health self-efficacy, and (RQ2) how does the use of mHealth technologies moderate the associations between the SDH status?

## 2. Materials and Methods

### 2.1. Data and Participants

Data were collected by means of a survey by a professional research company in South Korea, from 29 July to 3 August 2021. The research firm keeps a panel of more than 1.3 million individuals with proportionate representation of demographic characteristics of the South Korean population. The survey and consent to participate were approved by the Incheon National University Institutional Review Board (IRB No. 7007971–202106-003A). An e-mail invitation was sent to 10,322 panel members who had been chosen by the firm’s standardized protocol. Of those, 1952 panellists accessed the online survey site through the e-mail, and they were provided with brief information on the purpose of the survey and a consent from, which described the principles of voluntary participation, anonymity, and confidentiality. A total of 1227 respondents agreed to participate in the survey and completed it. The final sample size was 1187, excluding incomplete and potentially insincere responses. The average age of participants was 43.96 years (SD = 13.13, range: 20–69), and 49.1% were male. More than half of the participants (53.4%) had a bachelor’s degree, followed by less than high school (21.7%), some college or associate’s degree (16.1%), and graduate degree (8.8%) levels of education. Median monthly household income ranged from KRW 4.01 to 5.00 million. Table 1 presents descriptive statistics for the sample.

### 2.2. Measures

The measures were adapted from the Health Information National Trends Survey (HINTS 5, Cycle 1) [41]. HINTS is a national probability-based survey that has been regularly conducted by the US National Cancer Institute since 2003. The HINTS questionnaires are created to investigate American adults’ need for, access to, and use of health information and health-related behaviours [42]. In this study, the HINTS instrument served as a model to develop the health information trends survey for Koreans. A bilingual (Korean–English) translator translated the instrument from the source language (English) to the target language (Korean), and then another bilingual translator independently back-translated the instrument from the target language to the source language. Next, two experts in the field of health-survey research methods compared the two versions of the instrument to establish concept equivalence.

#### 2.2.1. Social Determinants of Health

In addition to the demographic and socioeconomic characteristics, social capital and healthcare quality were measured as social indicators of health. The demographic and socioeconomic factors included gender (mean = 1.51, SD = 0.50), education (mean = 2.49, SD = 0.93), and monthly household income (mean = 4.15, SD = 2.11). Social capital was measured using the following two questions: (1) “How many people can you count on to provide you with emotional support when you need it, such as talking over problems or helping you make difficult decisions?” and (2) “How many friends or family members do you have that you talk to about your health?” Responses were based on a 4-point Likert scale ranging from 1 = not at all to 4 = a lot (mean = 2.78, SD = 0.47, inter-item r = 0.62). To measure healthcare quality, respondents were asked to answer “Overall, how would you rate the quality of healthcare you received in the last 12 months?” Reponses were based on a 5-point Likert scale, ranging from 1 = poor to 5 = excellent (mean = 3.32, SD = 0.67).

#### 2.2.2. Use of mHealth Technologies

The use of mHealth technologies was assessed with the following two items: (1) “How often have you used health and wellness apps on your tablet or smartphone within the last 12 months?” and (2) “How often have you used a smart watch or wristband to monitor or take care of your health within the last 12 months?” Responses were based on a 5-point Likert scale ranging from 1 = not at all to 5 = always (mean = 2.02, SD = 0.98, inter-item r = 0.30).

#### 2.2.3. Health Outcomes

Health self-efficacy was assessed by a single item, asking participants to rate, “Overall, how confident are you about your ability to take good care of your health?” Responses were scored on a 4-point Likert scale, ranging from 1 = not confident at all to 4 = completely confident (mean = 2.55, SD = 0.56). Health status was measured by one item, asking respondents to evaluate their overall health condition using a 5-point Likert scale, ranging from 1 = poor to 5 = excellent (mean = 3.17, SD = 0.68).

### 2.3. Statistical Analyses

Table 2 provides bivariate correlations for the main variables. To examine the proposed hypotheses and research questions, two sets of hierarchical ordinary-least squares regression analyses were performed for the two dependent variables (i.e., health self-efficacy and health status). In each regression model, gender, education, monthly household income, social capital, and healthcare quality were set as the SDH in the first block, followed by the moderating variable, namely, the use of mHealth technologies, in the second block. Finally, five interaction terms were created and entered in the final block. An interaction term was formed by multiplying the standardized values for independent and moderating variables to remove multicollinearity problems between the interaction term and its components [43].

## 3. Results

### 3.1. Effects of Social Determinants on Health Outcomes

The hypotheses predicted that the SDH would be associated with health outcomes. As shown in Table 3, male respondents showed higher health self-efficacy (β = −0.14, *p* < 0.001) compared to female respondents, but there was no gender difference in health status. Thus, H1a was supported, but H1b was not. Participants with higher education levels reported superior health status (β = 0.07, *p* < 0.05) but not health self-efficacy. Thus, H2 was partially supported. Household income level, social capital, and healthcare quality were also positively associated with health outcomes. Specifically, individuals with higher household incomes, higher social capital, and higher healthcare quality showed higher levels of health self-efficacy (β = 0.09, *p* < 0.01 for household income; β = 0.29, *p* < 0.001 for social capital; β = 0.12, *p* < 0.001 for healthcare quality) and better self-reported health status (β = 0.08, *p* < 0.01 for household income; β = 0.21, *p* < 0.001 for social capital; β = 0.17, *p* < 0.001 for healthcare quality). These results supported H3, H4, and H5.

### 3.2. Moderating Effects of mHealth Technologies Usage

**Figure 2:**
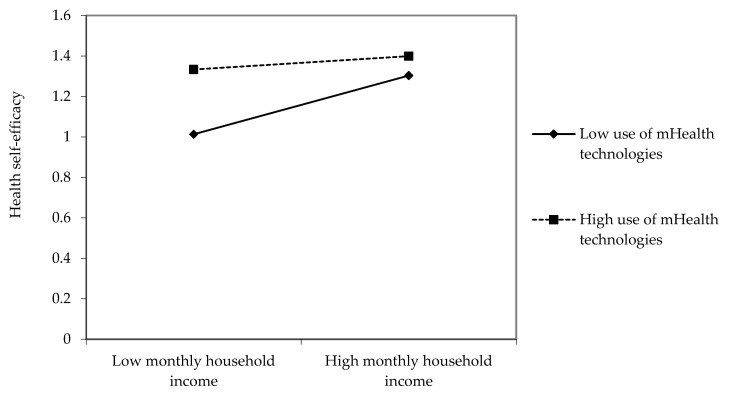
Interaction effects between monthly household income and use of mHealth technologies on health self-efficacy.

The research questions examined how the use of mHealth technologies would moderate the associations between the SDH and health outcomes. As presented in Table 3, four interactions were found to be significant. First, the positive association between monthly household income and health self-efficacy was weaker among high users of mHealth technologies than among low users of mHealth technologies (β = −0.06, *p* < 0.05, see Figure 2).

**Figure 3:**
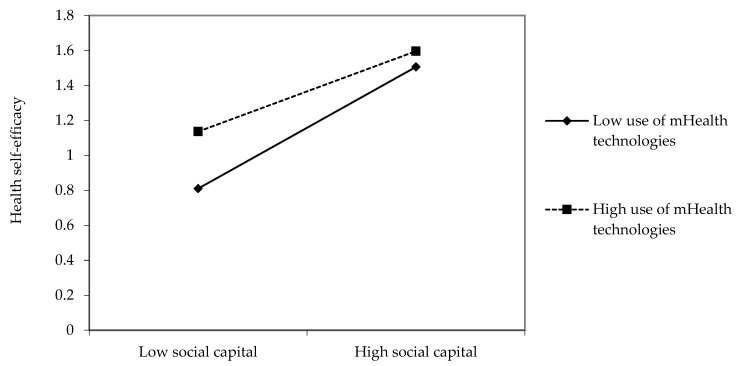
Interaction effects between social capital and use of mHealth technologies on health self-efficacy.

Second, the positive relationship between social capital and health self-efficacy was weaker among high users of mHealth technologies than among low users (β = −0.06, *p* < 0.05, see Figure 3).

**Figure 4:**
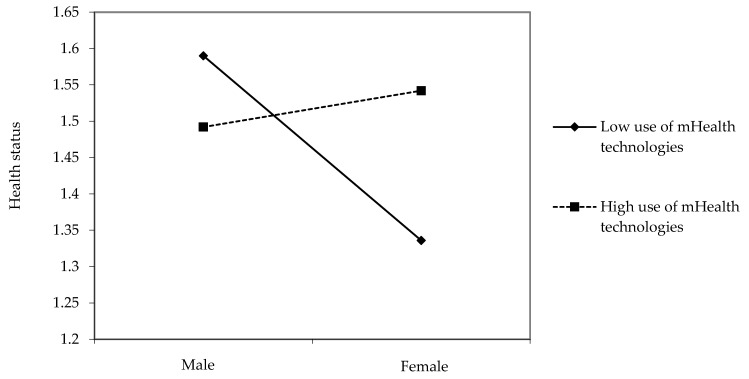
Interaction effects between gender and use of mHealth technologies on health status.

Third, mHealth technologies moderated the positive relationships between gender and health status. In the low-user group of mHealth technologies, men had better health status than women. In the high-user group, however, women reported better health status than men (β = 0.08, *p* < 0.01, see Figure 4).

**Figure 5:**
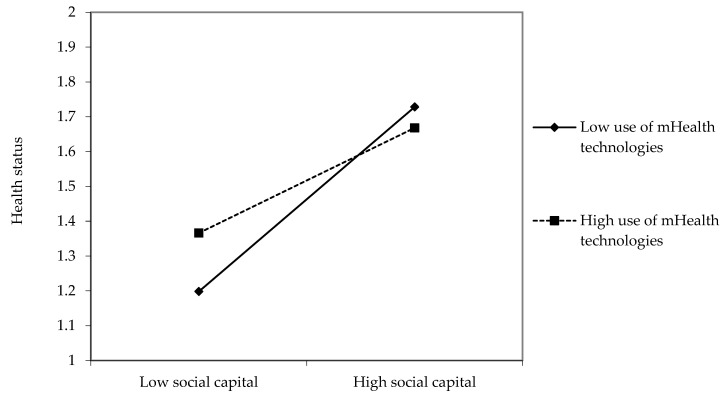
Interaction effects between social capital and use of mHealth technologies on health status.

Finally, the positive relationship between social capital and health status was weaker among high users of mHealth technologies than among low users of mHealth technologies (β = −0.06, *p* < 0.05, see Figure 5).

## 4. Discussion

This study examined the effects of the SDH on health self-efficacy and health status and how mHealth technologies moderate the impacts of the SDH on health outcomes. This study’s results yielded several significant findings regarding the impacts of the SDH on health self-efficacy and health status. The data showed that men had higher levels of health self-efficacy than women. Lee et al. [44] found that South Korean women showed worse self-reported health compared to U.S. women. South Korea’s traditional gender roles are likely to limit women’s time for rest and increase their stress level [45]. The higher an individual’s education level, the greater their health status. Household income, social capital, and healthcare quality were also found to be key drivers of health inequalities. Individuals with higher levels of monthly household income, social capital, and healthcare quality reported higher levels of health self-efficacy and greater health status. Using data of eight cohorts from Australia, the UK, Spain, the USA, Japan, South Korea, Mexico, and Europe, Wu et al. [46] examined the effects of education and wealth on health. They found that lower levels of education and wealth were related to poorer health and the strongest inequality trends for both education and wealth was found in the U.S. study.

The use of mHealth technologies moderated the effects of the SDH on health self-efficacy and health status. Neter et al. [33] revealed that online health-related activities were positively associated with a sense of empowerment and improved use of healthcare services. Similarly, this study showed positive relationships between mHealth technologies and health self-efficacy. However, the benefits of mHealth technologies were greater for the poor and disadvantaged populations. Specifically, monthly household income and social capital had weaker influences on health self-efficacy and health status for those who used higher levels of mHealth technologies. In other words, social and economic factors negatively influenced health self-efficacy and well-being among underserved populations, but mHealth technologies lessened the adverse effects of societal economic inequality on health. These results provide sufficient empirical evidence to support the contention that mHealth technologies hold great potential to reduce health inequalities and improve public health [23,24,25].

Given that mobile phones can be a more cost-effective way of accessing health information for those from a lower socioeconomic status [47], mHealth technologies can engage populations that have not been historically well served by the traditional health community. One explanation for this result is the ceiling or floor effect. Individuals with high income and significant social capital often already have high health self-efficacy and good health status. These individuals may have little space to accommodate the positive influence of mHealth. However, those with low incomes and little social capital can benefit from the use of mHealth technologies because they are likely to lack health self-efficacy or to have poor health.

The effects of gender on health status are different depending on the degree of use of mHealth technologies. In the high-user group, females reported better health status than males, and men showed better health status than women in the low-user group. The uses and gratification approach (UGA) [48] specifies what drives users to engage in regular intentional or routine usage of media. UGA indicates that men and women appear to exhibit very different motives for participation in physical activities. Previous studies have found that women are mostly motivated by appearance, weight management, and health factors, whereas strength, competition, and challenge are more important for men [49,50,51]. These different motives leading to physical activity are also seen in the gender-specific usage of mHealth technologies. Klenk et al. [52] found that enjoyment and achieving goals are the most important gratifications promoting engagement in a physical activity app, and women reported enjoyment and goal-setting to a greater degree than did men when using mHealth applications. This might reflect the higher motivation of women for health-oriented behaviour [53].

As a social determinant of health, the quality of medical care exerted a strong and direct impact on health outcomes, and mHealth technologies could not affect this influence. Although mHealth systems are sometimes presented as a replacement of traditional care, it is unlikely that they will completely replace traditional healthcare [54]. People want high-quality medical care first when they are sick. Inequalities in access to high- quality of healthcare services can accentuate inequalities in health. Another potential explanation is that the users of mHealth applications have difficulty in experiencing dynamic interactions with healthcare providers, which improve health outcomes. Most popular mHealth applications focus on consumer self-care [55]. Such applications are most used until the initial goals are met, and significantly decreased usage follows thereafter [56].

This study has substantive implications that should be noted. Among various SDH, this study highlighted socioeconomic factors that contribute to causing and reinforcing health inequalities. Poor health is not simply concentrated among those who are most deprived [57]. Health status declines with socioeconomic status, and thus it is important for public health researchers and policy makers to pay attention to the broader structure of socioeconomic conditions [58].

In addition to the main influence of the SDH on health, this research sheds light on the understanding of the moderating role of mHealth in the association between the SDH and health. Although the moderating influence of mHealth technologies showed very small effect sizes, the findings of this study indicate that mHealth technologies can potentially act as a buffer against the effects of the SDH, such as gender, household income, and social capital, which play a positive role in widening health disparities. Thus, it is critical and necessary to encourage underserved populations to adopt eHealth services and to provide them with timely and consistent access to mHealth technologies. To do so, public health researchers and practitioners should preferentially address the barriers to the use of mHealth among disadvantaged and underserved populations. There is a substantial body of literature that describes the significant challenges that exist to using mHealth technologies, including access to the Internet, acceptability of mHealth technologies, familiarity or knowledge of using mHealth apps, and digital health literacy [59,60,61]. In particular, the opportunities and challenges of mHealth have been evident in the promotion of health and well-being among the elderly during the COVID-19 outbreak. According to a systematic review of 10 studies on mHealth interventions for the health of older populations during the COVID-19 pandemic, mHealth services were used for treatment, health information provision, self-monitoring, and clinical consultations [62]. Meanwhile, the availability of mHealth devices, mobile Internet access, and the elderly’s skills and capabilities were barriers that should be addressed [62]. While COVID-19 could boost the development of digital health, thus affecting the quality of life, the digital divide could exacerbate health disparities across generations.

This study has some limitations that should be addressed in future research. First, it is based on a cross-sectional investigation. Although the hypotheses were derived from theoretical underpinnings and supported by empirical evidence, it was difficult to fully eliminate the possibility that the proposed associations among variables have the reversed causal relationship. For instance, social capital and perceived quality of healthcare can be influenced by health and well-being. Therefore, longitudinal studies with panel data are needed to rigorously examine the direction of causality among the theoretical variables. Second, the quota sampling used in this study may inhibit the generalizability of the findings. For example, compared to the general Korean population, the sample is highly educated, and this may bias the results. Future research should ideally employ probability sampling and nationally representative samples. There is the potential for bias in self-reports. Health self-efficacy and health status, which were used as the outcomes of interest in the analysis, are derived from the scales of the HINTS questionnaire. Although the survey instrument was constructed through a rigorous survey development and validation process, the measures based on a respondent’s recall likely produced measurement error [63]. For subjective measures in assessing health, future research should include objective measures of health, such as body mass index and obesity. Although social determinants of health are the social, economic, and environmental conditions that can influence health and well-being for older adults in particular, this study did not consider the significant role of age in examining social determinants of health. It is necessary to replicate these findings in the elderly population. Finally, the relatively small effect sizes of mHealth technologies should be noted. Besides mHealth, many factors have an influence on the health of individuals, thus limiting the potential impact of mHealth technologies.

## 5. Conclusions

Despite the limitations noted above, this study makes an important contribution to the literature by pointing to the SDH disparities and empirically demonstrating the effectiveness of mHealth technologies in reducing health disparities. To the extent that future studies support these findings, this work can support the strategies and efforts undertaken by health professionals and policymakers to mitigate health inequalities. This study demonstrates how mHealth technologies can contribute to addressing health inequalities and therefore may be of interest to individuals experiencing or concerned with health inequalities because of their social position.

## Figures and Tables

**Table 1:** Descriptive statistics of study participants.

	Participants (*N* = 1187)
Age (years)Mean (SD)	43.96 (13.13)
Gender	
Male	583 (49.1%)
Female	604 (50.9%)
Education	
High school or less	258 (21.7%)
Some college or associate’s degree	191 (16.1%)
Bachelor’s degree	634 (53.4%)
Graduate degree	104 (8.8%)
Monthly household income	
Less than 2.00 million Korean won ($1794 USD)	121 (10.2%)
2.01–3.00 million Korean won ($2691 USD)	176 (14.8%)
3.01–4.00 million Korean won ($3587 USD)	207 (17.4%)
4.01–5.00 million Korean won ($4484 USD)	218 (18.4%)
5.01–6.00 million Korean won ($5381 USD)	158 (13.3%)
6.01–7.00 million Korean won ($6278 USD)	102 (8.6%)
7.01–8.00 million Korean won ($7175 USD)	80 (6.7%)
8.01 or more Korean won	125 (10.5%)

**Table 2:** Bivariate correlations between main variables.

	1	2	3	4	5	6	7	8
1. Gender	1.00							
2. Education	−0.15 ***	1.00						
3. Monthly household income	0.04	0.22 ***	1.00					
4. Social capital	0.05	0.12 ***	0.18 ***	1.00				
5. Healthcare quality	−0.05	0.02	0.04	0.26 ***	1.00			
6. Use of mHealth technologies	−0.03	0.11 ***	0.13 ***	0.15 ***	0.17 ***	1.00		
7. Health self-efficacy	−0.13 ***	0.12 ***	0.14 ***	0.34 ***	0.21 ***	0.18 ***	1.00	
8. Health status	−0.05	0.12 ***	0.14 ***	0.27 ***	0.25 ***	0.11 ***	0.49 ***	1.00

*** *p* < 0.001.

**Table 3:** Results of hierarchical regression analyses of health outcomes.

	Health Self–Efficacy	Health Status
Block 1. Social determinants of health		
Gender (Male = 0)	−0.14 ***	−0.05
Education	0.05	0.07 *
Monthly household income	0.09 **	0.08 **
Social capital	0.29 ***	0.21 ***
Healthcare quality	0.12 ***	0.17 ***
∆*R^2^* (%)	0.158 ***	0.120 ***
Block 2. Moderator		
Use of mHealth technologies	0.10 ***	0.03
∆*R^2^* (%)	0.01 ***	0.001
Block 3. Interactions		
Gender × Use of mHealth technologies	0.00	0.08 **
Education × Use of mHealth technologies	0.02	0.00
Monthly household income × Use of mHealth technologies	−0.06 *	−0.03
Social capital × Use of mHealth technologies	−0.06 *	−0.06 *
Healthcare quality × Use of mHealth technologies	0.03	0.01
∆*R^2^* (%)	0.008 *	0.011 *
Total ∆*R^2^* (%)	0.177 ***	0.132 ***

Note. *N* = 1122. Cell entries standardized beta coefficient for Blocks 1 and 2, whereas cell entries are before-entry standardized beta coefficient for Block 3. *p*-Values for “∆*R*^2^” and “Total R” statistics result from F-change and F-test, respectively. * *p* < 0.05, ** *p* < 0.01, *** *p* < 0.001.

## Data Availability

The data used and/or analysed during the current study are available from the corresponding author on request.
